# Delayed‐Onset Cefepime Neurotoxicity in a Patient With Epilepsy and ESRD

**DOI:** 10.1155/carm/5491008

**Published:** 2026-07-03

**Authors:** Oluwadamilola Omojola, Sara Siddiqui, Veronica Zheng, Shannon Mccabe, Niti Girish Patel, Toluwalase Awoyemi, David Liebovitz

**Affiliations:** ^1^ Department of Internal Medicine, Feinberg School of Medicine, Northwestern University, Chicago, Illinois, USA, northwestern.edu; ^2^ Department of Internal Medicine, McGaw Medical Center, Northwestern University, Chicago, Illinois, USA, northwestern.edu

**Keywords:** cefepime, drug-induced encephalopathy, electroencephalography, end-stage renal disease, hemodialysis, neurotoxicity, seizures

## Abstract

Cefepime is associated with central nervous system toxicity, particularly in patients with renal impairment. Cefepime‐induced neurotoxicity (CIN) can manifest as seizures, encephalopathy, and other neurologic disturbances, often mimicking other conditions in complex patients. In this case report, we report a 42‐year‐old woman with end‐stage renal disease on hemodialysis, systemic lupus erythematosus, and epilepsy who presented with recurrent seizures after four months of indefinite cefepime therapy for chronic *Pseudomonas aeruginosa* catheter‐related bacteremia. Despite therapeutic clobazam levels and stable metabolic parameters, she experienced multiple seizure episodes during a dialysis session. Further investigation revealed characteristic electroencephalography (EEG) for cefepime neurotoxicity as well as elevated serum cefepime level. Following immediate cefepime discontinuation and transition to ceftazidime with optimization of antiepileptic therapy, the patient remained seizure‐free for the remainder of hospitalization. This case highlights the diagnostic challenge of cefepime neurotoxicity in patients with multiple comorbidities, particularly those with baseline seizure disorders and renal dysfunction. Clinicians should maintain a high suspicion for CIN in patients with renal impairment presenting with new or worsening seizures, utilize EEG findings and therapeutic drug monitoring when available, and promptly discontinue cefepime when toxicity is suspected.

**Key Clinical Message**
• Cefepime‐induced neurotoxicity (CIN) can occur after prolonged use, particularly in patients with renal impairment. In individuals with a history of epilepsy, new or clustered seizures should prompt consideration of drug‐induced causes, especially when therapeutic antiepileptic drug levels and a stable seizure control history are present. Electroencephalography (EEG) patterns suggestive of cefepime toxicity, in combination with elevated plasma drug levels, can support a timely diagnosis. Early recognition and withdrawal of cefepime are essential to prevent further neurologic deterioration. This case reinforces the importance of maintaining a high index of suspicion for cefepime neurotoxicity in complex patients receiving long‐term therapy.

## 1. Introduction

Cefepime, a fourth‐generation cephalosporin, is widely employed in treating severe bacterial infections, including those caused by *Pseudomonas aeruginosa*. Its broad‐spectrum activity and efficacy in critically ill patients have made it a mainstay in hospital formularies. However, its increasing use has also brought renewed attention to its potential for serious adverse effects, particularly neurotoxicity.

Among cephalosporins, cefepime is most notably associated with central nervous system toxicity, which can manifest as a spectrum of neurologic disturbances including seizures, myoclonus, aphasia, nonconvulsive status epilepticus, and encephalopathy [1]. Encephalopathy, in particular, may present with confusion, hallucinations, stupor, or coma, often mimicking other neurologic or metabolic conditions [1]. Cefepime neurotoxicity occurs in ∼1%–3% of treated patients and up to ∼15% in ICU populations [1, 2]. The proposed mechanism involves cefepime crossing the blood–brain barrier and exerting gamma‐aminobutyric acid (GABA) antagonism in a concentration‐dependent fashion [1]. Patients with impaired renal function are at particularly high risk due to decreased drug clearance and resultant accumulation [1, 3, 4].

Clinically, the recognition of cefepime‐induced seizures is challenging. In patients with baseline neurologic vulnerability, such as those with underlying epilepsy, metabolic derangements, or central nervous system infections, seizure activity is often attributed to more familiar causes. The absence of standardized diagnostic criteria for CIN further obscures timely diagnosis and intervention. Additionally, key questions remain unanswered: the precise toxic concentration of cefepime in the central nervous system, the full scope of mechanisms driving neurotoxicity, and the degree of risk in patients without overt renal dysfunction. Despite its clinical relevance, therapeutic drug monitoring remains rare, and long‐term neurologic outcomes in survivors of CIN are poorly characterized.

In this case report, we discuss the importance of maintaining a high index of suspicion for cefepime‐related seizures, particularly in complex clinical contexts.

## 2. Case History

The patient is a 42‐year‐old woman with end‐stage renal disease (ESRD) on hemodialysis who presented with recurrent seizures. During a routine dialysis session, she developed sudden right arm jerking and headache symptoms consistent with her prior seizure episodes. The dialysis was aborted approximately one‐third into the session. En route to the hospital, EMS reported multiple seizure‐like episodes. Upon arrival to the emergency department, she experienced two additional witnessed events: limb jerking, eyelid fluttering, and unresponsiveness to verbal and physical stimuli. Each episode resolved spontaneously within 2 minutes, with return to baseline mentation. Vital signs remained stable. Initial lactic acid was elevated at 3.1 mmol/L and normalized to 1.2 mmol/L. Lorazepam 2 mg IV was administered after the emergency department seizure.

Her history was notable for systemic lupus erythematosus (SLE) complicated by ESRD on thrice‐weekly hemodialysis, epilepsy managed with clobazam 10 mg nightly, and chronic *P. aeruginosa* bacteremia secondary to a right internal jugular dialysis catheter infection. Four months earlier, she had been hospitalized for *Pseudomonas* bacteremia, and due to limited vascular access and unstable housing that precluded peritoneal dialysis, the catheter was retained, and indefinite intravenous cefepime therapy was initiated per infectious disease (ID) recommendations at a fixed postdialysis dose of 2 g three‐times weekly, with each dose administered over 30 min. This regimen represented a renally appropriate dose per published hemodialysis dosing protocols [5]. Her last dose of cefepime was given 3 days prior to this seizure event that occurred during the dialysis session. The total duration of cefepime therapy prior to the seizure event was 4 months. Her last seizure had occurred 5 months prior, and an EEG at that time had shown no epileptiform activity.

**FIGURE 1:**
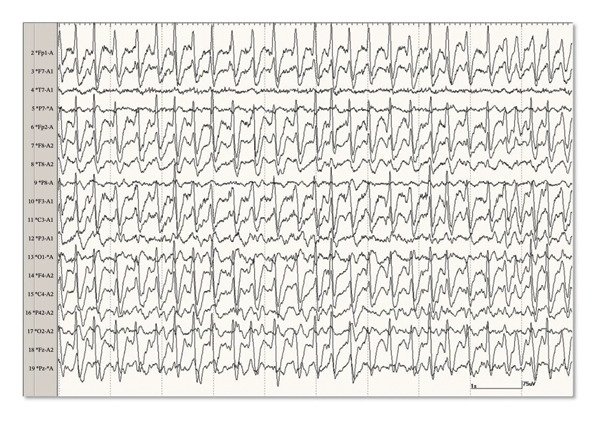
Representative EEG demonstrating generalized periodic discharges with triphasic morphology. The pattern is consistent with a toxic‐metabolic encephalopathy, and in this clinical context, is suggestive of cefepime‐associated neurotoxicity.

During this admission, neurology was consulted and recommended increasing the clobazam dose to 15 mg nightly. An EEG obtained early in the hospitalization demonstrated generalized periodic discharges (GPDs) with triphasic morphology and diffuse slowing, the findings consistent with CIN (Figure 1). A serum cefepime level from the day of admission was markedly elevated at 89 mg/L. Given the supratherapeutic level and the characteristic EEG findings, cefepime was promptly discontinued. In consultation with ID, she was transitioned to ceftazidime.

Following the discontinuation of cefepime and the adjustment of antiepileptic therapy, the patient remained seizure‐free for the remainder of her hospitalization. She was discharged in stable condition with plans for close outpatient neurology and ID follow‐up.

## 3. Discussion

This case illustrates the diagnostic complexity of evaluating seizures in a patient with multiple comorbidities, namely, ESRD, epilepsy, and chronic *Pseudomonas* bacteremia requiring indefinite cefepime therapy. The overlapping clinical manifestations of CIN, recurrent seizures, headaches, and encephalopathy can easily mimic breakthrough epileptic events, metabolic derangements, or uremic encephalopathy, particularly in patients with baseline neurologic disease. In such settings, CIN must remain a high‐priority differential, especially in the presence of renal impairment and chronic exposure to the drug. Several factors initially complicated the diagnostic picture. The patient had a known seizure disorder and was taking clobazam 10 mg nightly, without any missed doses. Hemodialysis, known to fluctuate antiepileptic drug levels and alter electrolyte balance, was also considered a potential contributor. However, the pattern of multiple seizures in close succession on the same day was atypical for the patient. Furthermore, a thorough metabolic workup showed no evidence of uremia or electrolyte abnormalities, and therapeutic drug monitoring confirmed clobazam levels were within the range. A turning point in the diagnostic evaluation was the identification of a markedly elevated cefepime level (89 mg/L), well above the threshold (> 20 mg/L) associated with neurotoxic symptoms [1, 4, 6, 7]. Approximately 68% of total body cefepime is removed during a 3‐h high‐flux dialysis session, necessitating postdialysis dosing [8]. Accounting for drug removal during the aborted dialysis session, the true cefepime trough in this case was presumably even higher. The temporal association between the seizures and cefepime use, combined with these supratherapeutic levels, was strongly suggestive of CIN. This is consistent with prior studies showing that 80%–90% of patients who develop CIN have underlying renal dysfunction [1, 4].

EEG played a critical role in supporting the diagnosis. In CIN, EEG abnormalities are common and include GPDs with triphasic morphology, moderate generalized slowing, and diffuse delta activity [7, 9–12]. In this patient, the EEG demonstrated continuous high‐amplitude alpha‐frequency activity (100–160 μV) with intermittent paroxysms of slow generalized waveforms (140–160 μV), which are characteristic of cefepime‐induced encephalopathy and helped distinguish CIN from primary epileptic activity.

### 3.1. Cefepime Pharmacology

Cefepime is a beta‐lactam with time‐dependent bactericidal activity, and its efficacy correlates with maintaining free drug concentrations above the organism’s minimum inhibitory concentration (MIC) for at least 60%–70% of the dosing interval for standard infections (with even higher goals for critically ill and septic patients). For patients with intermittent hemodialysis (iHD), cefepime concentrations fluctuate between doses and dialysis sessions, and high MIC organisms or harder to penetrate sites of infection may require higher post‐HD doses or supplemental doses between sessions. These pharmacokinetic and pharmacodynamic (PK/PD) targets must be balanced with the risk of side effects and individualized dosing should be based on the organism MIC, site of infection, and dialysis modality. In this case, the *Pseudomonas* susceptibility for cefepime was reported using a Kirby–Bauer disk diffusion which classifies antibiotics as susceptible (S), intermediate (I), or resistant (*R*) and does not report an exact MIC [13]. For *Pseudomonas,* a disk diffusion susceptible report could span a range of MICs from 1 mg/L to ≤ 8 mg/L, making it tricky to formulate an individualized dose [14].

For patients on iHD, the FDA package insert recommends administering cefepime at a dose of 500–1000 mg every 24 h, with doses given post‐HD on dialysis days. Alternatively, three‐times weekly, postdialysis dosing allows for severe infections to be treated on an outpatient basis and avoids the need for additional vascular access [8]. Evidence supports this dosing strategy as well‐tolerated and effective in achieving PK/PD goals with the option to dose cefepime at 1000–1500 mg after dialysis for highly susceptible pathogens and 1500–2000 mg after dialysis for less susceptible pathogens, such as *Pseudomonas* [15, 16]. Importantly, the major safety concern in ESRD is drug accumulation leading to neurotoxicity, including encephalopathy, aphasia, myoclonus, and nonconvulsive status epilepticus [16]. Risk is significantly higher in renal impairment, with up to a 10‐fold increase when doses are not adjusted or kidney function acutely declines. The U.S. Food and Drug Administration Drug Safety Communication highlighted cases of nonconvulsive status epilepticus associated with cefepime, particularly in patients with renal dysfunction who did not receive appropriate dose adjustments, emphasizing the need for careful renal dosing, close neurologic monitoring, and prompt discontinuation if toxicity is suspected [17]. While our patient’s cefepime dosing was appropriately adjusted for renal function, neurotoxicity can occur even with appropriate renally adjusted dosing. However, previously reported cases of neurotoxicity in patients who had received appropriate renal dose adjustment exists, underscoring that adherence to recommended dosing does not entirely eliminate this risk and emphasizes the importance of close monitoring in this population [17, 18].

### 3.2. Management

Management of this patient centered on immediate discontinuation of cefepime, substitution with ceftazidime dosed 1 g thrice weekly posthemodialysis, and escalation of clobazam from 10 mg to 15 mg nightly to reinforce seizure prophylaxis. Accumulation of ceftazidime can also cause neurotoxicity, particularly in renal impairment and advanced age, though it is less frequently reported in the literature [3, 19]. Compared to ceftazidime, cefepime has greater affinity for GABA‐A receptors; its antagonism at these receptors lowers the seizure threshold. Close monitoring of patients with risk factors for neurotoxicity is still warranted while receiving ceftazidime therapy [20]. After these interventions, the patient experienced no further seizures, highlighting the potential for rapid clinical improvement once the offending agent is withdrawn. Therapeutic drug monitoring, while not yet standard practice, may have utility in preventing toxicity, particularly in patients receiving long‐term cefepime with compromised renal function.

This case is particularly notable for the diagnostic challenge in distinguishing CIN from breakthrough seizures in a patient with established epilepsy. Delayed onset of neurotoxic symptoms after months of cefepime therapy, is not unexpected given the pharmacokinetics and the expected accumulation of cefepime in ESRD. CIN can develop insidiously after prolonged, previously well‐tolerated use, especially in patients with renal dysfunction or underlying neurologic disease [21]. The case also illustrates the diagnostic pitfalls in attributing recurrent seizures to epilepsy alone in such patients. In this context, early consideration of CIN, EEG evaluation, and drug level measurement can expedite diagnosis and prevent further neurologic decline.

Despite growing recognition of CIN, several knowledge gaps persist. The precise toxic thresholds for neurotoxicity are not well defined, and there is limited risk‐stratification guidance for patients outside the renal impairment spectrum. Additionally, standardized diagnostic criteria and the role of routine cefepime level monitoring remain unresolved. Future studies are needed to define safe plasma concentration thresholds, establish evidence‐based monitoring protocols, and improve recognition of atypical presentations in vulnerable populations.

## 4. Conclusion

CIN should be high on the differential in patients with renal impairment who present with new or worsening seizures, especially those with a known seizure disorder. This case highlights that neurotoxicity can develop after prolonged cefepime use and may mimic breakthrough epilepsy. Clinicians must recognize that therapeutic antiepileptic drug levels do not exclude cefepime toxicity. Prompt identification, EEG interpretation, and drug discontinuation are critical to avoid delays in care and prevent serious neurologic sequelae.

## Author Contributions

Oluwadamilola Omojola contributed to case conceptualization, data collection, literature review, manuscript drafting, and figure creation.

Sara Siddiqui contributed to the clinical care of the patient, provided input on case interpretation, and reviewed and edited the manuscript for intellectual content.

Veronica Zheng contributed to the clinical care of the patient, provided input on case interpretation, and reviewed and edited the manuscript for intellectual content.

Toluwalase Awoyemi: contributed to the clinical care of the patient, provided input on case interpretation, reviewed and edited the manuscript for intellectual content, and approved the final version for submission.

Niti Girish Patel provided pharmacological expertise on cefepime dosing, pharmacokinetics, and neurotoxicity; contributed to case interpretation; and reviewed and edited the manuscript for intellectual content.

Shannon McCabe provided pharmacological expertise on cefepime dosing, pharmacokinetics, and neurotoxicity; contributed to case interpretation; and reviewed and edited the manuscript for intellectual content.

David Liebovitz oversaw clinical care, provided guidance on case framing, and critically reviewed and edited the manuscript for intellectual content.

## Funding

This research did not receive any specific grant from funding agencies in the public, commercial, or not‐for‐profit sectors.

## Ethics Statement

Ethics approval was not required for this case report in accordance with the policies of our institutional review board and national ethical guidelines for single‐patient case reports.

## Consent

Verbal informed consent was obtained from the patient for the publication of this case report and any accompanying images or data.

## Conflicts of Interest

The authors declare no conflicts of interest.

## Data Availability

All data relevant to this case report are included within the article. No additional data are available.
